# The Study of Caspian Roach (*Rutilus caspicus*) Fry Health Fed with Phytobiotic-Supplemented and Salinity Stress Resistance with Emphasis on Gill Tissue Pathology

**DOI:** 10.1155/2023/4581144

**Published:** 2023-08-11

**Authors:** Allieh Bairami Igdery, Mohammad Farhangi, Hossein Adineh, Hojatollah Jafaryan, Zia Kordjazi, Seyed Hossein Hoseinifar

**Affiliations:** ^1^Department of Fisheries, Faculty of Agriculture and Natural Resources, Gonbad Kavous University, Gonbad Kavous, Golestan, Iran; ^2^Department of Fisheries, Faculty of Fisheries and Environmental Sciences, Gorgan University of Agricultural Sciences and Natural Resources, Gorgan, Iran

## Abstract

Herbal treatment augments immune and antioxidant responses and suppresses stress in fish. Ginger (*Zingiber officinale*) is a popular plant with medicinal uses because of its immunostimulant, antimicrobial, and antioxidant characteristics. This study aimed to investigate the effects of ginger (*Z. officinale*) on growth, digestive enzymes activity, antioxidant and immune response, and salinity stress resistance of Caspian roach (*Rutilus caspicus*). Fish (0.98 ± 0.09 g) were divided into four treatment-fed diets containing 0 (control, Z0), 10 (Z10), 20 (Z20), and 30 (Z30) g/kg ginger powder for 56 days and then subjected to 2 g/L salinity stress for 48 hr. The highest final weight of specific growth rate (SGR), superoxide dismutase (SOD), and catalase activity and the lowest final weight of feed conversion ratio (FCR) and malondialdehyde (MDA) were observed in fish-fed diets containing 10 and 20 g/kg ginger inclusion. Intestinal protease activity significantly increased in Z10 treatment, and the highest amylase and lipase activities were related to control, including 10 g/kg ginger in the diet compared to the control group had a significant effect on immune indices such as immunoglobulin M (IgM) and ACH50 (*p* < 0.05). The highest lysozyme was obtained in Z20 treatment, which had a significant difference in the control (*p* < 0.05). Cortisol and glucose levels were significantly lower in ginger treatments than the control before and/or after salinity stress. Histopathologic results showed that hyperplasia, edema, expansion of secondary lamella, epithelial cells, and necrosis of gills were most common lesions. However, the results of this study demonstrate that using ginger powder in addition to improving of growth, it can be also effective in survival rate of Caspian roach fingerling as an endangered species exposed to salinity stress.

## 1. Introduction

The Caspian roach (*Rutilus caspicus*) is one of the most economical fish of the Caspian Sea. However, overfishing and the destruction of spawning grounds have led to a decline in the wild population and put this species on the brink of extinction. Based on the International Union for Conservation of Nature classification, this species is classified as an endangered species [1]. The importance of the population of Caspian roach and other fish in terms of the survival of the Caspian Sea ecosystem has made it necessary to investigate the increase of their production efficiency. Based on quantitative development in the farming of aquatic animals, fish farming has confronted many problems, such as changes in water quality, adverse environmental factors, the outbreak of diseases, and nutritional problems. The outbreak of diseases and environmental stress (including salinity stress) as the main problem of aquaculture have affected the development of this industry in many countries [2]. Salinity is one of the stressful and influencing factors on metabolism and aquatic animals' distribution, which affects existing growth and development processes. The survival of the animal in an environment depends on its osmotic ability to exposure to the salinity fluctuations of the living environment. In contrast, the growth of aquatic animals is influenced by various environmental and nutritional factors [3].

Herbal food additives can affect the growth rate in aquatic animals by affecting indicators such as digestibility, feeding efficiency, and food taste. Medicinal plants can activate defense mechanisms, specific or nonspecific defense in fish [4, 5]. These substances are vital as medicinal agents for disease control. Ginger (*Zingiber officinale*) is one of the medicinal plants used in aquatic food. This plant has compounds such as calcium, phosphorus, sodium, potassium, iron, chromium, magnesium, cobalt, zinc, selenium, mercury, chlorine, bromine, fluorine, rubidium, scandium, selenium, thiamin, riboflavin, niacin, pyridoxine, vitamins (A, B, C, E), fatty oil, spicy compounds, resins, proteins, cellulose, pentose, and starch [6, 7]. One of the distinctive features of ginger is its natural antioxidant properties (gingerols, shogaols, and zingerone) [8, 9]. This plant is known as an effective agent on the digestive and immune systems in humans and animals (including fish) [10].

Behavioral reactions are an indicator of aquatic sensitivity to stress and gills are sensitive to environmental stress due to direct contact with the aquatic environment. So, in ecotoxicological research, behavior is a valuable end point as a result of physiological changes affected by environmental pollutants [11, 12]. Therefore, histopathological changes in gills can be used as a marker to evaluate the effects of salinity on fish. Chloride cells in the epithelium of the gills are the most ion exchange cells, and environmental salinity fluctuations change the structure of these cells [13]. Since the fish are in direct contact with the surrounding aquatic environment, changes in the physicochemical parameters of the water such as salinity affect the physiological response of the fish [14]. Several studies have been conducted on the positive effect of ginger and other herbal supplements on the activity of the digestive system and the growth rate of fish [8, 15–21]. But so far, no research has been done regarding the improvement of the physiological conditions of the Caspian roach fed with ginger to salinity stress resistance. Nowadays, some research has investigated the role of food supplements in increasing the resistance of fish to various environmental, bacterial, and especially toxic stresses [22–25]. So, this study aimed to investigate the effectiveness of ginger on the resistance and safety of Caspian roach in confronting salinity stress.

## 2. Materials and Methods

### 2.1. Fish Preparation and Laboratory Conditions

Six hundred Caspian roach with an average weight of 0.98 ± 0.09 g were obtained from Sijowal (Golestan, Iran) and transferred to the fisheries laboratory of Gonbad Kavous University. They were acclimated to experimental conditions for 14 days, and then randomly stored in 12 tanks (water volume, 50 L) at a density of 50 fish per tank. The fish were stored in four treatments with three replicates.

Ginger (*Z. officinale*) powder was purchased from the herbal store (Gonbad Kavous, Iran). The most important compounds of ginger powder were used, which were prepared from a medicinal plant store (Table 1) [26–28]. The basal diet was formulated by mixing the feedstuffs and adding with 250 ml water per kg (Table 2). The obtained dough was dried through a meat grinder and then cut into appropriate sizes using a fan. The experimental diets were prepared similarly but added 0 (Z0), 10 (Z10), 20 (Z20), and 30 (Z30) g/kg ginger powder (replaced with wheatmeal). The concentrations used were based on previous studies [21, 29].

The feeds were stored at −20°C until use [30]. Analysis of the proximate composition of diets was performed according to the Association of Official Analytical Chemists [31] standard. The fish were fed the above diets at 3% of biomass within 8 weeks.

### 2.2. Growth Performance and Nutrition

After feeding for 56 days, the fish were fasted for 24 hr to measure biomass and take samples. Growth and feed parameters were calculated based on the following equations at the end of the test period [32]:

(1) $$\begin{array}{c} \text{Weight gain rate }\left(\mathrm{WGR},\%\right)=100\times \frac{\left(\text{Final weight}-\text{Initial weight}\right)}{\text{Initial weight}}, \end{array}$$

(2) $$\begin{array}{c} \text{Specific growth rate }\left(\mathrm{SGR},\%/\mathrm{day}\right)=100\times \frac{\left(\ln\text{final weight}-\ln\text{ initial weight}\right)}{\text{Days}}, \end{array}$$

(3) $$\begin{array}{c} \text{Feed conversion ratio }\left(\mathrm{FCR},\;g/g\right)=\frac{\mathrm{Dry}\text{ feed intake}}{\left(\text{Final weight}-\text{Initial weight}\right)}, \end{array}$$

(4) $$\begin{array}{c} \text{Food conversion efficiency }\left(\mathrm{FCE},\%\right)=\left[\frac{\left(\text{Final weight}-\text{Initial weight}\right)}{\mathrm{Dry}\text{ feed intake}}\right]\times 100, \end{array}$$

(5) $$\begin{array}{c} \text{Survival rate }\left(\%\right)=100\times \frac{\text{Final number }\mathrm{of}\text{ fish}}{\text{Initial number }\mathrm{of}\text{ fish}}. \end{array}$$

### 2.3. Intestinal Sampling and Measurement of Digestive Enzymes

Eighteen fish from each treatment were randomly caught at the end of the feeding period. The intestine was separated from the intra-abdominal part and frozen in liquid nitrogen for analysis. The samples were homogenized and centrifuged at 25,000 rpm for 20 min and then the supernatants were used for analysis [30]. Intestinal amylase activity was measured using 0.3% soluble starch as a substrate through the method presented by Langlois et al. [33]. Lipase activity was measured by hydrolysis of *p*-nitrophenyl myristate as substrate by the method of Iijima et al. [34]. Protease activity was measured based on the instructions of Walter [35] using a 1% weight–volume ratio of casein (*w*/*v*) as substrate.

### 2.4. Salinity Stress

Fish fed with ginger powder were kept in direct exposure to the salinity stress resistance of the Caspian roach for 48 hr. For this purpose, 75 fish in each treatment (25 fish in each replication) were stored in 25 L tanks with 12 g/L salinity. Then, the mortality rate was recorded during 48 hr. The sampling of fish mucus was done in two stages (after 56 days feeding with different levels of ginger powder and after 48 hr of salinity stress). Skin mucus samples were randomly prepared from 30 fish in each treatment (10 fish from each tank) and after centrifugation, the mucus samples were stored at −80°C.

## 3. Fish Health Assessment

Mucus collection was done naturally by the method provided by Guardiola et al. [36]. Briefly, skin mucus was collected by gently rubbing the dorsolateral surface of the fish in an anterior-to-caudal direction in less than 2 min [14]. Finally, the mucus was separated by a centrifuge for 10 min at 3,000 rpm at a temperature of 4°C. The samples were stored in a 2 ml microtube and kept in a freezer at −80°C until the mucus immunoassay was performed [36]. Alternative hemolytic complement activity (ACH50) was determined following the method described by Sunyer and Tort [37] based on the hemolysis of rabbit red blood cells (RaRBCs) [38]. Mucus total protein (TP), glucose, and alkaline phosphatase (ALP) were determined by Parsazmon's kits (Parsazmon Company, Iran) according to the company's protocol. The protease activity of mucus was measured based on the azocasein hydrolysis method [39]. Lysozyme enzyme was measured by turbidity method using a spectrophotometer [40]. Total immunoglobulin M (IgM) was measured by the method of Siwicki and Anderson [41]. The cortisol level of the skin mucus was measured by the competitive ELISA method using a commercial kit (IBL Co., Gesellschaft für Immunchemie und Immunbiologie).

### 3.1. Antioxidant Ensymes

Muscle superoxide dismutase (SOD) activity was determined detailed in the method of McCord and Fridovich [42]. Catalase activity was evaluated following the method of Aebi [43]. The level of malondialdehyde (MDA) was measured using a colorimetric method as an indicator of lipid peroxidation [44].

### 3.2. Gill Histology

After salt stress, 25 fish were randomly caught from each treatment and anesthetized with cloves. Gill tissue samples were examined histopathologically after fixation with 10% formalin [45].

### 3.3. Data Analysis

First, the data normality was determined by the Shapiro–Wilk and Levene tests. It was analyzed using one-way analysis of variance (ANOVA) and then Duncan's multiple range test to compare the means between groups at the 95% confidence level (*p* < 0.05). Two-way ANOVA was also used to test the effects of the diet, salinity stress, and their interactions. Statistical analyses were performed by SPSS version 21 and Excel 2019 software.

## 4. Results

**Figure 1:**
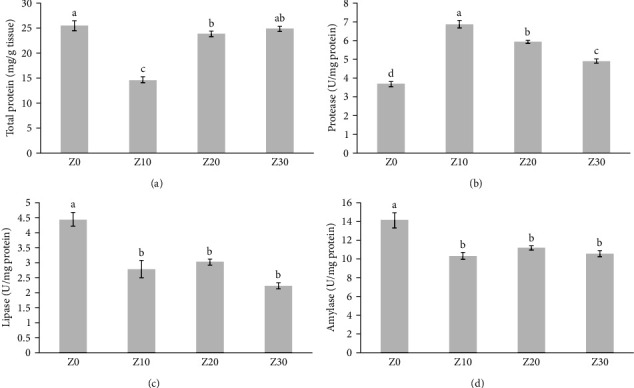
Mean ± *SD* of digestive enzymes activities of Caspian roach fed 10–30 g/kg (Z10, Z20, and Z30) dietary ginger (*Zingiber officinale*) powder and control without additives (Z0) for 56 days (mean ± *SD*, *n* = 3): (a) total protein, (b) protease, (c) lipase, and (d) amylase. Different lowercase letters within a column show significant effects of the treatments (*p* < 0.05).

The growth performance of fish fed to ginger powder for 8 weeks is shown in Table 3. Final weight, weight gain, and SGR have significant differences between experimental treatments (*p* < 0.05). The highest growth parameters were observed in Z10 and Z20 treatments, and the lowest growth parameters were observed in Z0 and Z30 treatments. There was no significant difference in FCR between experimental treatments (*p* > 0.05). Digestive enzymes in Caspian roach fed with different levels of ginger powder are presented in Figure 1. The obtained results showed that the highest total protein, lipase, and amylase were in control, which were significantly increased compared to other experimental treatments (*p* < 0.05). The highest and lowest protease was obtained, respectively, in the treatments containing Z10 treatments (6.86 ± 0.20 units/mg protein) and the control treatment (3.67 ± 0.15 units/mg protein), which had a significant difference (*p* < 0.05).

**Figure 2:**
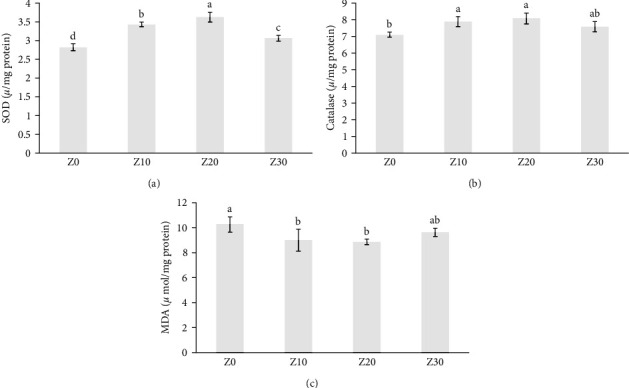
Mean ± *SD* of antioxidant enzymes: SOD, superoxide dismutase (a); catalase (b); and MDA, malondialdehyde (c) of Caspian roach fed 10–30 g/kg (Z10, Z20, and Z30) dietary ginger (*Zingiber officinale*) powder and control without additives (Z0) for 56 days (mean ± *SD*, *n* = 3). Different lowercase letters within a column show significant effects of the treatments (*p* < 0.05).

Antioxidant enzymes in fish fed with different levels of ginger powder are presented in Figure 2. SOD and catalase enzymes significantly increased in Z20 treatment compared to the control. The highest MDA was in the control treatment (*p* < 0.05). Immune factors in the fish mucus fed with ginger powder (before stress) and salinity stress (after stress) are shown in Table 4. Immunoglobulin concentration and ACH50 activity significantly increased in Z10 compared to other experimental treatments (*p* < 0.05). Also, the protease, total protein, and lysozyme activity increased significantly in Z20 (*p* < 0.05). After salinity stress for 48 hr, the highest values of immunoglobulin and lysozyme concentrations were obtained in the nutritional treatments and the lowest in the control treatment. The highest protease, protein, and ACH50 were obtained in Z20 (*p* < 0.05). The total immunoglobulin, ACH50, and lysozyme activity had a significant decrease after salinity stress (*p* < 0.05). The mucus stress indices in fish before and after stress are shown in Table 5. Cortisol and glucose significantly decreased in the Z10 treatment and increased in the Z0 treatment before salinity stress and the end of the feeding period (*p* < 0.05). The cortisol and glucose increased in experimental treatments after salinity stress. At the end of the feeding, ALP increased significantly in the control treatment (*p* < 0.05). The concentration of this enzyme was the highest in the control treatment and the lowest in the Z20 treatment after salinity stress.

**Figure 3:**
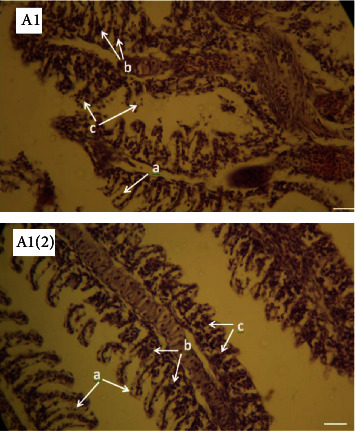
Photomicrographs of gill in the fish (Z0) exposed to salinity stress (H&E, ×400). A1: Arrows show (a) edema and detachment of covering epithelium in secondary lamellae, (b) basal hyperplasia in secondary lamellae, and (c) necrosis in covering cells. A1(2): Arrows show (a) edema and detachment of covering epithelium in secondary lamellae, (b) basal hyperplasia in secondary lamellae, and (c) sticking attach secondary lamellae.

**Figure 4:**
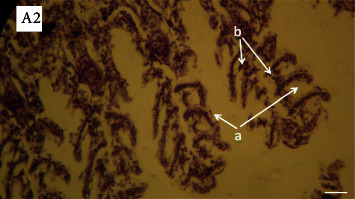
Photomicrographs of gill in the fish (Z10) exposed to salinity stress (H&E, ×400). A2: Arrows show hemorrhage: (a) edema and detachment of covering epithelium in secondary lamellae and (b) basal hyperplasia in secondary lamellae.

**Figure 5:**
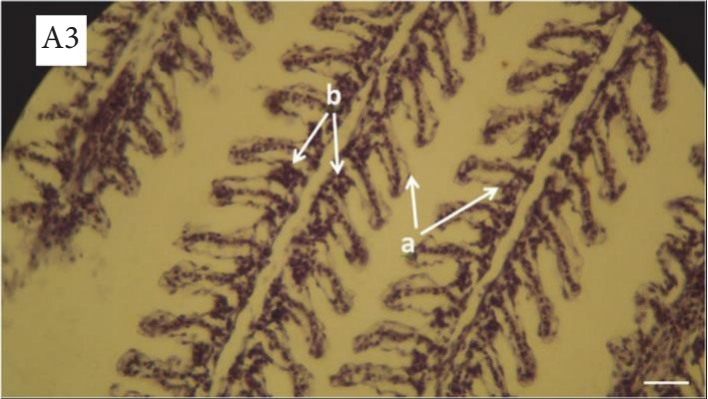
Photomicrographs of gill in the fish (Z20) exposed to salinity stress (H&E, ×400). A3: Arrows show hemorrhage: (a) edema and detachment of covering epithelium in secondary lamellae and (b) basal hyperplasia in secondary lamellae.

**Figure 6:**
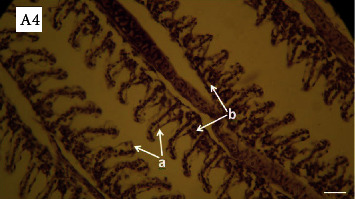
Photomicrographs of gill in the fish (Z30) exposed to salinity stress (H&E, ×400). A4: Arrows show hemorrhage: (a) edema and detachment of covering epithelium in secondary lamellae and (b) basal hyperplasia in secondary lamellae.

The lesions mainly observed after salinity stress in gills included edema, basal hyperplasia in secondary lamellae, detachment of covering epithelium in secondary lamellae, and necrosis in covering cells. Lesions were observed in all investigated groups (Figures 3–6). However, the percentage of lesions was different in fish fed with ginger (especially Z3 treatment).

## 5. Discussion

Aquaculture is one of the most effective methods for preserving threatened and depleted aquatic species and closing the supply–demand gap for aquatic seafood. The aquatic environment, diet, and farmed stock are three interrelated factors that affect aquaculture productivity [46, 47]. The foundation of sustainable aquaculture is improving these factors. Nowadays, using herbal supplements have a significant role in increasing the growth and resistance of fish against various diseases. The increase in weight of fish fed with diets containing growth and immune system stimulants can increase the level of health, improve the digestion and absorption of food, or stimulate the secretion of digestive enzymes [48, 49]. Optimizing dietary factors can lead to ecological adaptation, better growth, and reduced heavy losses during fish rearing [5]. The results of ginger powder on fish growth factors showed that adding ginger powder caused a significant increase in the growth rate of this fish. Based on the results of Abbasi Ghadikolaei et al. [8], the maximum final weight, weight gain, SGR, and FCR were obtained in the treatment fed with ginger powder. These results are related to the function of the active substance zingerone in ginger, its role in accelerating the activities of digestive enzymes in the intestinal wall, and increasing the absorption of consumed feed. Shaluei et al. [50] investigated the effect of ethanolic ginger extract on the growth performance and skin mucosal immune parameters of rainbow trout. Yousefi et al. [51] reported that using ration containing black seed had significantly increasing effects on the growth and resistance of exposed common carp to glyphosate poison. Dadgar et al. [48] showed that using 0.5% garlic powder in the diet improved the growth indices of *Carassius auratus*. The results of similar studies show the positive performance of ginger powder and other medicinal plants as growth stimulants and immunity of fish [52]. Generally, digestion is a key process in animal metabolism that determines the availability of nutrients for biological activities. Therefore, it is necessary to study it to find out about the dietary conditions and the adaptation of organisms to changes in diet [53]. The analysis of digestive enzymes of Caspian roach fed with different levels of ginger powder showed that the highest total protein, amylase, and lipase were obtained in the control treatment, which was significantly increased compared to other experimental treatments (*p* < 0.05). The results of various researches show the effectiveness of plant extracts on digestibility and increase in digestive enzymes [54–56]. Shakya [55] reported that using a diet containing herbal supplements increased the digestive enzymes. In this study, the performance of ginger powder was positive only on the protease enzyme and no increase was observed in other digestive enzymes. Ginger increases metabolic activity in the body and stimulates protease enzymes to break down proteins and convert them into small peptides and amino acids. Digestive enzymes increase the efficiency of food utilization. Therefore, using various supplements that stimulate digestive enzymes will play an effective role in improving the growth and nutrition of fish [53]. Herbal supplements used in fish diets can act as an appetizing agent and stimulate digestive enzymes [4, 48, 49].

The antioxidant system is an indicator of fish health and is responsible for protecting aquatic animals against oxidative stress [9, 57]. The oxidative status has higher levels in fish fed with ginger powder. Fazelan et al. [29] observed that ginger supplementation significantly increased the antioxidant parameters of common carp. Zargar et al. [58] found improvement in antioxidant status in rainbow trout with ginger powder supplementation. Proper management to reduce stress on farmed fish is one of the goals of fish farmers for more production. Using medicinal plants can be effective in reducing the stress caused to fish [59]. In the present study, the mucus stress indices had significant before and after stress. Although the cortisol and glucose increased in experimental treatments after salinity stress, the lowest was observed in the Z20 treatment and the highest in the Z0 treatment. Similar results have been published for the Caspian roach salinity stress resistance by adding fructooligosaccharide [60], garlic [61], earthworm extract [62], and savory essential oil to the diet [63] and rearing in the biofloc system [14]. ALP concentration increased significantly in the control treatment and decreased in the Z30 treatment at the end of the feeding period (before stress) (*p* < 0.05). The concentration of this enzyme after stress with seawater was the highest in the control treatment and the lowest in the Z20 treatment. ALP is a multifunctional enzyme involved in membrane transport activities [64, 65]. ALP in fish exposed to salinity increased significantly. The increase in the level of these enzymes is due to cytolysis and the leakage of enzymes in the immune system, which indicates tissue damage in organs such as the liver and kidney [66].

Hematological changes are generally investigated to determine the health status against environmental, disease, and nutritional stress [67]. Fish are sensitive and vulnerable to diseases, environmental, and nutritional stresses; their resistance depends entirely on the nonspecific immune system [49]. Therefore, it is necessary to improve the nonspecific immune system of fish. The lysozyme is an immune protective enzyme that plays an important role in the immune status of aquatic animals. Therefore, a decrease in the levels of lysozyme and immunoglobulin stress can reduce health and increase the mortality rate of fish [49]. Immune factors of mucus in fish fed with ginger powder showed that immunoglobulin concentration and ACH50 activity increased significantly in the Z10 treatment compared to the control treatment. Also, the protease, total protein, and lysozyme in the Z20 treatment had a significant increase compared to other experimental treatments (*p* < 0.05). The lysozyme and immunoglobulin concentration, ACH50 activity had a significant decrease after salinity stress. Several studies emphasize the importance of using medicinal plants to improve the status of nonspecific immune factors such as lysozymes [49, 51, 68]. Karimi Pashaki et al. [49] reported that 5% of garlic extract had the greatest effect on the amount of lysozyme and IgM, which indicates the effect of this medicinal plant on strengthening the nonspecific immune system of carp. Yousefi et al. [51] reported that using black seeds in the common carp diet led to more appropriate levels of fish blood serum factors such as total protein, albumin, cholesterol, and triglyceride. Shaluei et al. [50] reported the level of skin mucosa lysozyme, ALP, and protease activity in treatments containing ginger extract (2.5 and 5 g) had a significant increase compared to the control.

Gills are sensitive to environmental stress due to direct contact with the aquatic environment. Therefore, histopathological changes in gills and other organs (such as the liver and kidney) can be used as an indicator in evaluating the effects of various types of stress in fish (especially salinity). Chloride cells in the epithelium of the gills are the most important cells for ion exchange, and the structure of these cells changes with changes in the salinity of the environment [13]. Since fish are in direct contact with the surrounding aquatic environment, changes in the physicochemical parameters of water such as salinity affect them. Lesions such as hyperemia, bleeding, hyperplasia of the tip of gill filaments, edema, and expansion of secondary lamellae were seen in all groups exposed to salt stress and their levels were different in fish fed with ginger. Other studies have proven the effects of various pollutants and environmental stress on gill tissue [69–71]. Beikzadeh et al. [72] investigated the effect of salinity stress on chloride cells and gill histopathology of common carp fingerlings fed with edible cortisol. The investigated damages (hyperplasia, hypertrophy, hyperemia, and edema) were more in the control group than in the cortisol-treated fish, and fewer damages were observed in the cortisol-treated fish. The number of chloride cells increased with salinity stress, which could be due to the better performance of the osmotic regulation of these fish against salinity stress. Heidari et al. [73] reported that simultaneous and gradual changes in salinity and temperature on the gill tissue and chloride cells of common carp increased the number and area of chloride cells. Basir and Peyghan [74] reported on the effect of different environmental salinities on gill chloride cells in common carp, that the highest number of chloride cells in the filament position was related to 8 ppt salinity and the lowest number in the control treatment.

## 6. Conclusion

Ginger powder as a plant antioxidant compound has the property of improving growth performance, antioxidant capacity, immunity, histopathological of gill tissue, and antistress effects to salinity in Caspian roach. Based on the results, 100–200 g/kg of *Z. officinale* is recommended for food supplements of *R. caspicus* and it is expected that using ginger powder in the diet of the Caspian roach will increase their survival in the breeding process of this valuable species.

## Figures and Tables

**Table 1:** The most important compounds of ginger powder (*Zingiber officinale*) [26–28].

Compounds	Area (%)
*β*-Phellandrene	16.07
*α*-Farnesene	14.26
*α*-Muurolene	11.57
Camphene	6.45
*β*-Sesquiphellandrene	5.68
*α*-Bisabolene	4.46
Geranial	4.2
Zingiberene	2.4
Zingiberenol	3.5
*α*-Pinene	3.03
*β*-Pinene	2.4
Myrcene	2.39
Curcumene	2.09

**Table 2:** Ingredients and proximate composition (g/kg diet on dry matter basis) of the experimental diets containing 10–30 g/kg ginger (*Zingiber officinale*) powder.

Ingredients	Control	Z10	Z20	Z30
Fish meal^a^	125	125	125	125
Meat meal^b^	220	220	220	220
Soybean meal	230	230	230	230
Wheatmeal	340	330	320	310
Fish oil	8	8	8	8
Soybean oil	8	8	8	8
Corn flour	54	54	54	54
L-Lysine^c^	5	5	5	5
L-Methionine	5	5	5	5
Vitamin premix^d^	2.5	2.5	2.5	2.5
Mineral premix^e^	2.5	2.5	2.5	2.5
*Zingiber officinale*	0	10	20	30
Proximate analysis				
Dry matter	86.19	85.30	84.41	83.52
Crude protein (%)	37.61	37.47	37.32	37.18
Crude fat (%)	6.36	6.34	6.32	6.30
Crude fiber	2.72	2.69	2.67	2.65
Crude ash (%)	6.31	6.29	6.27	6.24

*Note*: ^a^Pars Kilka Co., Mazandaran, Iran (Kilka powder analysis; protein: 70%–72%, fat: 8%–11%, ash: 11.6%, moisture: 7%–9%). ^b^Makianmehr Co., Golestan, Iran. ^c^Morghenojan Co., Tehran, Iran. ^d^Vitamin premix (per kg of diet): vitamin A, 2,000 IU; vitamin B1 (thiamin), 5 mg; vitamin B2 (riboflavin), 5 mg; vitamin B6, 5 mg; vitamin B12, 0.025 mg; vitamin D3, 1,200 IU; vitamin E, 63 mg; vitamin K3, 2.5 mg; folic acid, 1.3 mg; biotin, 0.05 mg; pantothenic acid calcium, 20 mg; inositol, 60 mg; ascorbic acid (35%), 110 mg; and niacinamide, 25 mg. ^e^Mineral premix (per kg of diet): MnSO_4_, 10 mg; MgSO_4_, 10 mg; KCl, 95 mg; NaCl, 165 mg; ZnSO_4_, 20 mg; KI, 1 mg; CuSO_4_, 12.5 mg; FeSO_4_, 105 mg; and Co, 1.5 mg.

**Table 3:** Growth parameters of Caspian roach fed 10–30 g/kg (Z10, Z20, and Z30) dietary ginger (*Zingiber officinale*) powder and control without additives (Z0) for 56 days (mean ± *SD*, *n* = 3).

Growth parameters	Treatments			
	Z0	Z10	Z20	Z30
Initial weight (g)	1.02 ± 0.09	0.94 ± 0.12	1.00 ± 0.07	0.96 ± 0.08
Final weight (g)	1.85 ± 0.21^bc^	1.99 ± 0.28^ab^	2.09 ± 0.26^a^	1.76 ± 0.22^c^
Body weight (%)	81.17 ± 12.90^b^	115.33 ± 42.55^a^	109.24 ± 29.64^a^	83.41 ± 24.97^b^
Specific growth rate (%/day)	1.05 ± 0.13^b^	1.33 ± 0.34^a^	1.30 ± 0.25^a^	1.06 ± 0.24^b^
Feed conversion ratio	3.50 ± 0.69	2.95 ± 0.93	2.93 ± 0.82	3.71 ± 1.02
Food conversion efficiency (g/cm^3^)%	29.53 ± 5.45^ab^	36.70 ± 10.88^a^	36.25 ± 8.97^ab^	28.89 ± 7.97^b^
Survival (%)	100	100	100	100

*Note*: Different lowercase letters within a row show significant effects of the treatments (*p* < 0.05).

**Table 4:** Skin mucus immune parameters of Caspian roach fed 10–30 g/kg (Z10, Z20, and Z30) dietary ginger (*Zingiber officinale*) powder and control without additives (Z0) for 56 days (mean ± *SD*, *n* = 3).

Skin mucus immune parameters	Treatments					Two-way ANOVA		
	Z0 (control)		Z10	Z20	Z30	Stress	Nutriton	Reaction
Total protein (g/dl)	Before stress	100.32 ± 1.14^c–D^	106.70 ± 8.39^bc−CD^	131.31 ± 5.87^a–A^	113.61 ± 3.45^b–C^	*p* < 0.001	*p* < 0.001	*p* < 0.001
	After stress	44.27 ± 1.30^c–F^	51.41 ± 0.55^b–EF^	123.61 ± 4.51^a–B^	54.16 ± 0.88^b^			

Protease (U/mg)	Before stress	57.42 ± 1.42^b–BC^	69.79 ± 1.25^ab–AB^	82.37 ± 1.78^a–A^	64.45 ± 21.24^ab–BC^	*p* = 0.077	*p* < 0.0001	NS
	After stress	52.42 ± 0.48^d–C^	55.43 ± 1.46^c–BC^	81.21 ± 0.83^a–A^	61.48 ± 1.16^b–BC^			

Lysozyme (U/ml/min)	Before stress	24.69 ± 0.53^b–BC^	25.29 ± 0.46^b–B^	28.58 ± 0.64^a–A^	25.47 ± 0.96^b–B^	*p* < 0.001	*p* < 0.001	*p* = 0.002
	After stress	20.72 ± 0.47^b–D^	24.54 ± 1.59^b–BC^	23.71 ± 0.29^a–C^	23.46 ± 0.95^a–C^			

ACH50 (%)	Before stress	128.34 ± 0.81^b–B^	131.57 ± 0.67^a–A^	130.67 ± 0.53^a–A^	129.04 ± 0.54^b–B^	*p* < 0.001	*p* < 0.001	*p* = 0.009
	After stress	122.40 ± 0.58^b–E^	123.69 ± 0.59^bc–D^	125.78 ± 0.59^a–C^	123.23 ± 0.83^b–B^			

Total immunoglobulin (mg/dl)	Before stress	11.00 ± 0.20^c–D^	15.28 ± 0.67^a–A^	13.50 ± 0.33^b–B^	12.90 ± 0.05^b–DE^	*p* < 0.001	*p* < 0.001	NS
	After stress	5.52 ± 0.20^d–H^	9.62 ± 0.26^a–E^	7.40 ± 0.33^b–F^	6.48 ± 0.05^c–G^			

*Note*: TP, total protein; IgM, immunoglobulin M; ANOVA, analysis of variance. The small letter indicates the comparison of the average of experimental treatments separately (at the end of the feeding period) and (after salinity stress). Capital letters indicate the comparison of the average of all treatments with each other (end of nutrition and end of salinity). Therefore, two small and uppercase letters are displayed for each number (*p* < 0.005). NS in a row shows no significant effects of the treatments (*p* < 0.005).

**Table 5:** Mean ± *SD* of stress parameters of Caspian roach fed 10–30 g/kg (Z10, Z20, and Z30) dietary ginger (*Zingiber officinale*) powder and control without additives (Z0) for 56 days (mean ± *SD*, *n* = 3).

Stress parameters	Treatments					Two-way ANOVA		
	Z0 (control)		Z10	Z20	Z30	Stress	Nutriton	Reaction
Cortisol (ng/ml)	Before stress	4.79 ± 0.09^a–C^	3.66 ± 0.17^c–E^	4.28 ± 0.14^b–D^	3.88 ± 0.18^c–E^	*p* < 0.001	*p* < 0.001	*p* < 0.001
	After stress	5.76 ± 0.22^a–A^	5.22 ± 0.13^b–B^	4.58 ± 0.32^c–CD^	5.35 ± 0.26^ab–B^			

Glucose (mg/dl)	Before stress	17.03 ± 0.74^a–B^	11.50 ± 0.43^c–E^	16.18 ± 1.01^ab–BC^	15.30 ± 1.10^b–CD^	*p* < 0.001	*p* < 0.001	*p* < 0.001
	After stress	20.43 ± 0.76^a–A^	17.02 ± 1.28^b–B^	13.81 ± 0.53^c–D^	21.60 ± 1.00^a–A^			

Alkaline phosphatase (U/L)	Before stress	59.53 ± 0.45^b–B^	42.72 ± 1.37^b–CD^	40.30 ± 2.24^Bc–DE^	38.63 ± 1.87^c–E^	*p* = 0.026	*p* < 0.001	*p* < 0.001
	After stress	73.96 ± 1.15^a–A^	33.70 ± 2.69^c–F^	22.54 ± 0.68^d–G^	44.32 ± 1.51^b–C^			

*Note*: The small letter indicates the comparison of the average of experimental treatments separately (at the end of the feeding period) and (after salinity stress). Capital letters indicate the comparison of the average of all treatments with each other (end of nutrition and end of salinity). Therefore, two small and uppercase letters are displayed for each number (*p* < 0.05).

## Data Availability

The raw data supporting the conclusions of this article will be made available by the authors, without undue reservation.
